# The Impact of Adjunct Hyperbaric Oxygen Therapy on Healing Diabetic Foot Ulcers: A Retrospective Case-Control Study at a Tertiary Care Center

**DOI:** 10.7759/cureus.99432

**Published:** 2025-12-17

**Authors:** Lloyson Benix, Mangalanandan T Sukumaran, Vivek Lakshmanan, Ravi Sankaran, Vijay Kumar

**Affiliations:** 1 Surgery, KS Hegde Medical Academy, Mangaluru, IND; 2 Podiatry, Amrita School of Medicine, Amrita Institute of Medical Sciences and Research, Amrita Vishwa Vidyapeetham, Kochi, IND; 3 Physical Medicine and Rehabilitation, Amrita School of Medicine, Amrita Institute of Medical Sciences and Research, Amrita Vishwa Vidyapeetham, Kochi, IND; 4 Public Health Dentistry, Amrita School of Dentistry, Amrita Institute of Medical Sciences and Research, Amrita Vishwa Vidyapeetham, Kochi, IND

**Keywords:** diabetic foot infection, diabetic foot ulcer (dfu), diabetic peripheral neuropathy (dpn), hyperbaric oxygen therapy (hbot), ischemic limb, nonhealing wound, peripheral arterial diseases, surgical wound dressing, ­wound healing, wound healing time

## Abstract

Background: A reduced oxygen supply to diabetic foot ulcers slows down wound healing, as it affects angiogenesis, reepithelialization, and extracellular matrix formation. A compromised blood supply impairs oxygen delivery to the wound, creating a hypoxic environment around it. Hyperbaric oxygen therapy (HBOT) increases the concentration of dissolved oxygen in the plasma. It compensates for the existing hypoxia and promotes wound healing. HBOT promotes neovascularization and matrix formation, decreases inflammation, and increases reactive oxygen free radicals, which can kill bacteria. Moreover, HBOT overwhelms the enzymes that reduce free radical production. Adverse effects are very rare when patients are carefully screened before treatment. However, HBOT cannot maintain the increased oxygen concentration. Therefore, a discussion of the overall benefits of HBOT for wound healing is warranted. This study aimed to evaluate the effect of HBOT on the healing of diabetic foot ulcers in terms of the time taken for complete wound healing.

Methods: A retrospective case-control study was conducted at a tertiary care center on 235 inpatients selected according to defined inclusion and exclusion criteria. The patients’ clinical parameters influencing wound healing, such as age, HbA_1_c levels, serum albumin, hemoglobin, ankle-brachial index, and comorbid conditions, were recorded. The severity of wounds according to the University of Texas wound classification system was recorded. Patients who received standard-of-care treatment were included in the control group. Patients who received HBOT as an adjunct therapy along with standard of care treatment were included in the experimental group. All patients in both groups underwent necessary surgical and vascular interventions. Wound follow-up was performed for six months, and complete wound healing was the primary endpoint.

Results: Logistic regression showed that the HBOT group patients had improved wound healing (odds ratio (OR) = 2.84) compared with the control group patients. The logistic regression analysis indicated that the HBOT group was associated with a significant improvement in wound healing after accounting for the severity of diabetic foot ulcers. HBOT was predominantly used for patients with worse baseline ulcers, and the median time to healing was significantly longer in the HBOT group than in the control group. Potential confounders of the association between HBOT and wound healing were identified using a Bayesian network. Even after adjusting for potential confounders, HBOT showed a favorable trend (OR = 2.32), although it did not reach statistical significance.

Conclusion: This study demonstrates a positive correlation between HBOT and the healing of diabetic foot ulcers. HBOT, when used as an adjunct to standard-of-care treatment, significantly shortened the time to healing in University of Texas grade 1 ulcers. Further research in this area may justify considering HBOT as a standard-of-care treatment for diabetic foot ulcers.

## Introduction

Inflammation, proliferation, and remodeling are the three overlapping stages of wound healing. Impaired vasculature hinders the transport of oxygen to the wound, which creates a hypoxic environment around the wound immediately following the triggering event. The hypoxia is exacerbated by the recruitment of inflammatory cells with high oxygen consumption. Long-term oxygen deprivation in chronic wounds hinders the healing process by inhibiting angiogenesis, reepithelialization, and extracellular matrix formation [1].

HBOT and its mechanism of action

Treatment with 100% oxygen at levels higher than atmospheric pressure is known as hyperbaric oxygen therapy (HBOT). While the treatment chamber's pressure is raised to more than 1 atmosphere absolute (ATA), the patient only intermittently breathes 100% oxygen. An increase in dissolved oxygen in the plasma due to an increase in the partial pressure of arterial oxygen causes hyperoxygenation, which is an application of Henry's law. Six milliliters of O₂ are dissolved for every 100 mL of plasma at a pressure of 3 ATA, providing an O₂ delivery capacity equivalent to that of hemoglobin-bound O₂ [2].

During collagen synthesis, oxygen is essential for the hydroxylation of lysine and proline residues as well as for collagen cross-linking and maturation. HBOT reduces hypoxia, resulting in the synthesis of sufficient quantities of mature collagen. Angiogenesis is greatly aided by hypoxia, but sufficient tissue oxygen concentration is necessary for the formation of an adequate capillary network for complete wound healing. HBOT increases the oxygen gradient between the center and periphery of the wound, causing significant angiogenic stimulation, which results in increased neovascularization and fibroblast proliferation [2].

Benefits of HBOT

As oxygen cannot be stored in tissues, daily HBOT is required to provide an adequate oxygen supply to the site of injury to promote wound healing progression from the inflammatory phase to the proliferative phase. Increased oxygen concentration leads to increased production of reactive oxygen and nitrogen species, which play important roles in signaling pathways that promote neovascularization and matrix formation and reduce inflammation [3]. These free radicals cause DNA damage and stop bacteria from metabolizing by oxidation of proteins and lipids in membranes. Enzymes such as superoxide dismutase, catalase, glutathione, and glutathione reductase prevent the generation of free radicals until the oxygen load overwhelms these enzymes. Moreover, HBOT facilitates the oxygen-dependent peroxidase system, which is used by leukocytes to kill bacteria and is especially efficient against anaerobic bacteria that do not possess superoxide dismutase [2].

Limitations of HBOT

Previous research has demonstrated that the adverse effects of HBOT in accredited facilities with skilled staff are typically mild and bearable. Because of the relatively safe technique and proper pretreatment screening of patients, serious adverse effects are rare. A study by Zhang et al. showed that HBOT is more likely to cause adverse reactions when the chamber pressure is above 2 ATA [4]. Cardiovascular effects, oxygen poisoning, barotrauma, hypoglycemia, and confinement anxiety are the adverse effects to consider during screening [3]. The oxygen concentration in poorly vascularized wounds rapidly drops after treatment. Therefore, the therapeutic effectiveness of HBOT is considered uneven and inadequate. The inability of HBOT to ensure a continuous, adequate oxygen supply to the wound is the primary cause of this performance [5].

Study objective

The present study aimed to evaluate the effect of HBOT on the healing of diabetic foot ulcers by assessing the time taken for complete wound healing.

## Materials and methods

**Figure 1:**
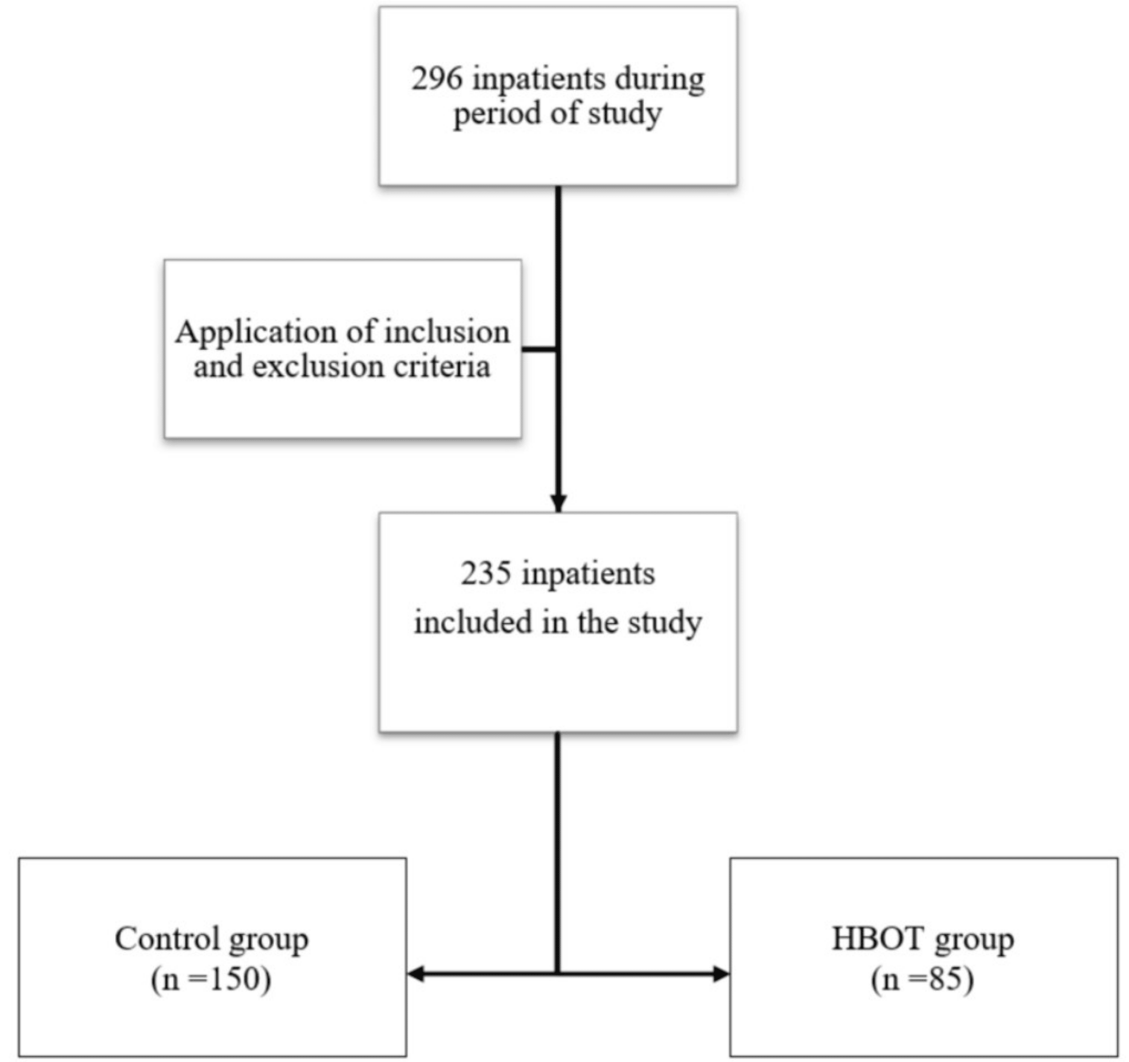
CONSORT flow diagram of the study population HBOT: hyperbaric oxygen therapy; CONSORT: Consolidated Standards of Reporting Trails

This was a retrospective case-control study conducted at the Amrita Institute of Medical Sciences, a tertiary care hospital in Kochi, India. Of the 296 inpatients admitted to the Department of Podiatry between August 2022 and August 2024, 235 inpatients were included in the study according to the inclusion and exclusion criteria (Figure 1).

The inclusion criteria were patients over the age of 18 years with foot ulcers of any University of Texas grade, glycated hemoglobin (HbA1c) levels more than 6.5, and ankle-brachial index (ABI) more than 0.4. Exclusion criteria were patients who were lost to follow-up within six months of treatment; patients who underwent local oxygen therapy; noncompliant patients; and those with contraindications for HBOT, such as malignancy, pregnancy, seizures, middle ear problems, claustrophobia, congenital spherocytosis, or severe obstructive pulmonary disease.

Certain clinical parameters of the patients were examined, such as age, HbA1c, serum albumin, hemoglobin, and ABI, and comorbid conditions, such as diabetes mellitus, hypertension, chronic kidney disease (CKD), coronary artery disease (CAD), chronic liver disease (CLD), diabetic retinopathy, and diabetic neuropathy. The presence of infection and ischemia in the wound was also recorded, and the wounds were graded using the University of Texas grading and staging system.

Patients with Stage A wounds had neither infection nor ischemia; those with Stage B wounds exhibited only infection; those with Stage C wounds had only ischemia; and those with Stage D wounds had both infection and ischemia. Grade 0 wounds were completely epithelialized pre- or postulcerative lesions; Grade 1 wounds were superficial ulcers not involving tendon, capsule, or bone; Grade 2 wounds were ulcers penetrating to tendon or capsule; and Grade 3 wounds were ulcers penetrating to bone or joint. Of the 235 patients, 150 and 85 were included in the control group and case group, respectively.

Control definition

Of the inpatients who were selected on the basis of inclusion and exclusion criteria, those who had received standard-of-care dressings with appropriate medical and surgical management were included in the control group. Standard-of-care dressings included moist saline dressings with antiseptic, silver alginate, or hydrocolloid dressings.

Case definition

Of the inpatients selected as per inclusion and exclusion criteria, those who were diagnosed with peripheral vascular occlusive disease on the basis of ABI less than 0.9, transcutaneous oxygen pressure values less than 40, or Doppler signals showing reduced flow were included in the case (experimental) group. They had undergone multiple sessions of HBOT in addition to receiving standard-of-care dressings and appropriate medical and surgical management. The HBOT protocol was an average of 15 sessions, with one session per day. HBOT was administered for six days per week with a break of one day before the next session. Each session lasted approximately 1.5 hours, and the pressure ranged between 2 and 3 ATA. All patients were compliant with the treatment protocol.

In both groups, oral or intravenous antibiotics were administered, and surgical interventions such as debridement and minor or major amputations were performed if deemed necessary. Moreover, vascular interventions, such as peripheral angioplasty or bypass surgery, were performed if deemed necessary. Negative pressure wound therapy was also used when appropriate, and well-granulated wounds were covered with partial-thickness skin grafts. Clinical progress during the first six months of follow-up was recorded, and ulcer healing within six months of the initial assessment and treatment was considered the primary outcome.

Statistical analysis 

Continuous variables were summarized using mean and standard deviation, whereas categorical variables were summarized using contingency tables. Categorical variables were compared using Pearson's chi-square test and Fisher's exact test, and continuous variables were compared using the Wilcoxon rank-sum test. A Bayesian network was constructed using the R package MXM (R Foundation for Statistical Computing, Vienna, Austria) to identify potential confounding variables influencing the primary outcome of complete wound healing within six months. In this probabilistic graphical model, nodes represented clinical and demographic variables, and edges denoted conditional dependencies between them. The network identified infection, ischemia, CAD, CLD, neuropathy, and retinopathy as key confounders associated with delayed wound healing. These variables were then included in a multivariable logistic regression model to adjust for group imbalances and more accurately estimate the effect of HBOT. Using the Bayesian network, the study leveraged a data-driven approach to confounder selection, enabling a more robust assessment of causal relationships in this nonrandomized retrospective dataset. Logistic regression was performed to analyze the effect of HBOT on wound healing, adjusting for the University of Texas grading for wound severity. Additionally, logistic regression was used to assess the relationship between the groups adjusted for the confounders identified by the Bayesian network. A significance level of α = 0.05 was used for all analyses. All statistical analyses were conducted using R software (version 4.4.1).

## Results

A total of 296 patients were admitted to the Department of Podiatry between August 2022 and August 2024. After applying the inclusion and exclusion criteria, 235 inpatients were included in the final analysis, as shown in Figure 1. The baseline characteristics of the 235 patients included in the study, comparing the control group (n = 150) with the HBOT case group (n = 85), are presented in Table 1.

**Table 1:** Baseline characteristics of patients in the control and HBOT groups and wound classification ^1^Median (Q1, Q3); n (%) ^2^Wilcoxon rank-sum test; Pearson's chi-square test; Fisher's exact test POVD: peripheral occlusive vascular disease; CKD: chronic kidney disease; CLD: chronic liver disease; CAD: coronary artery disease; HBOT: hyperbaric oxygen therapy

Characteristic	Total (n = 235)^1^	Control (n = 150)^1^	HBOT (n = 85)^1^	Statistic^2^	p value^2^
Age (years)	64 (57, 71)	66 (57, 75)	62 (57, 67)	3.43	0.002
HbA_1_c	8.60 (7.50, 10.30)	8.10 (7.40, 10.60)	8.70 (7.90, 10.20)	-1.42	0.4
Hypertension	144 (61%)	86 (57%)	58 (68%)	2.28	0.10
POVD	84 (36%)	36 (24%)	48 (56%)	23.51	<0.001
CKD	46 (20%)	36 (24%)	10 (12%)	4.41	0.023
CLD	16 (6.8%)	13 (8.7%)	3 (3.5%)	1.52	0.13
CAD	71 (30%)	52 (35%)	19 (22%)	3.34	0.048
Retinopathy	20 (8.5%)	17 (11%)	3 (3.5%)	3.30	0.039
Nephropathy	11 (4.7%)	8 (5.3%)	3 (3.5%)	0.095	0.8
Neuropathy	46 (20%)	45 (30%)	1 (1.2%)	26.83	<0.001
University of Texas grade					
0A	2 (0.9%)	0 (0%)	2 (2.5%)	-	-
0B	1 (0.4%)	1 (0.7%)	0 (0%)	-	-
0C	2 (0.9%)	0 (0%)	2 (2.5%)	-	-
0D	1 (0.4%)	0 (0%)	1 (1.2%)	-	-
1A	16 (6.9%)	8 (5.3%)	8 (9.9%)	-	-
1B	18 (7.8%)	15 (10%)	3 (3.7%)	-	-
1C	3 (1.3%)	2 (1.3%)	1 (1.2%)	-	-
1D	11 (4.8%)	5 (3.3%)	6 (7.4%)	-	-
2A	4 (1.7%)	1 (0.7%)	3 (3.7%)	-	-
2B	32 (14%)	30 (20%)	2 (2.5%)	-	-
2C	1 (0.4%)	0 (0%)	1 (1.2%)	-	-
2D	23 (10.0%)	16 (11%)	7 (8.6%)	-	-
3A	11 (4.8%)	3 (2.0%)	8 (9.9%)	-	-
3B	37 (16%)	35 (23%)	2 (2.5%)	-	-
3C	5 (2.2%)	1 (0.7%)	4 (4.9%)	-	-
3D	64 (28%)	33 (22%)	31 (38%)	-	-
Infection	187 (81%)	135 (90%)	52 (64%)	21.07	<0.001
Ischemia	110 (48%)	57 (38%)	53 (65%)	14.79	<0.001
Time to heal (in months)	4.0 (3.0, 6.0)	3.0 (3.0, 6.0)	6.0 (4.0, 9.0)	42.97	<0.001

As shown in Table 1, there were some imbalances in the patient characteristics between the two groups. The HBOT group patients were significantly younger than the control group patients (p = 0.002), as shown by the median ages of 62 and 66 years, respectively. Ischemia was significantly more frequent in the HBOT group (n = 53, 65%) than in the control group (n = 57, 38%; p < 0.001). Peripheral occlusive vascular disease (POVD) was also more prevalent in the HBOT group (n = 48, 56%) than in the control group (n = 38, 24%; p < 0.001). HBOT had been preferred for patients with ischemia and POVD, hence the above-mentioned significance.

In contrast, CKD was more common in the control group (n = 36, 24%) than in the HBOT group (n = 10, 12%; p = 0.023). CAD and retinopathy also showed statistically significant differences between the groups (p = 0.048 and p = 0.039, respectively), with a higher frequency in the control group. Notably, neuropathy was significantly more prevalent in the control group (n = 45, 30%) than in the HBOT group (n = 1, 1.2%; p < 0.001). Long-term diabetes predisposes individuals to neuropathy, CAD, nephropathy, and retinopathy, and neuropathy is a primary risk factor for infection. Thus, a higher age and higher frequency of neuropathy, CAD, CKD, retinopathy, and infection were observed in the control group.

Furthermore, the severity of diabetic foot ulcers, as classified by the University of Texas grade, differed between the groups, with most of the University of Texas grade 3 ulcers being present in the HBOT group. This suggests that the HBOT group had a higher proportion of patients with severe wounds, which is consistent with the possibility that HBOT is predominantly used for patients with worse baseline ulcers. Therefore, the median time to healing was significantly longer in the HBOT group than in the control group (six and three months, respectively; p < 0.001). The healing rate at six months in the control and HBOT groups was 81.2% and 65.3%, respectively. This finding can be partially attributed to the smaller sample size of the HBOT group compared with that of the control group.

The results of the logistic regression analysis that examined the impact of HBOT on wound healing, while accounting for ulcer severity assessed using the University of Texas wound classification system, are presented in Table 2. The analysis revealed that patients with grade 1 ulcers had significantly higher odds of wound healing (odds ratio (OR) = 41.5; 95% confidence interval (CI) = 3.14-1,065, p = 0.006). However, the effect of grade 2 and 3 ulcers on wound healing was not statistically significant (p = 0.3 and p = 0.8, respectively). Notably, after adjusting for wound severity, the HBOT group demonstrated a statistically significant improvement in wound healing in terms of healing time compared with the control group (OR = 2.84, 95% CI = 1.44-5.86, p = 0.003). The logistic regression indicated that the HBOT group was associated with a significant improvement in wound healing when accounting for the severity of diabetic foot ulcers.

**Table 2:** Logistic regression of the effect of HBOT on wound healing while adjusting for wound severity according to the University of Texas wound classification system OR: odds ratio; CI: confidence interval; HBOT: hyperbaric oxygen therapy

Characteristic	OR	95% CI	p value
University of Texas grade			
0	-	-	-
1	41.5	3.14, 1,065	0.006
2	2.47	0.30, 15.4	0.3
3	1.20	0.16, 6.89	0.8
Group			
Control	-	-	-
HBOT	2.84	1.44, 5.86	0.003

**Figure 2:**
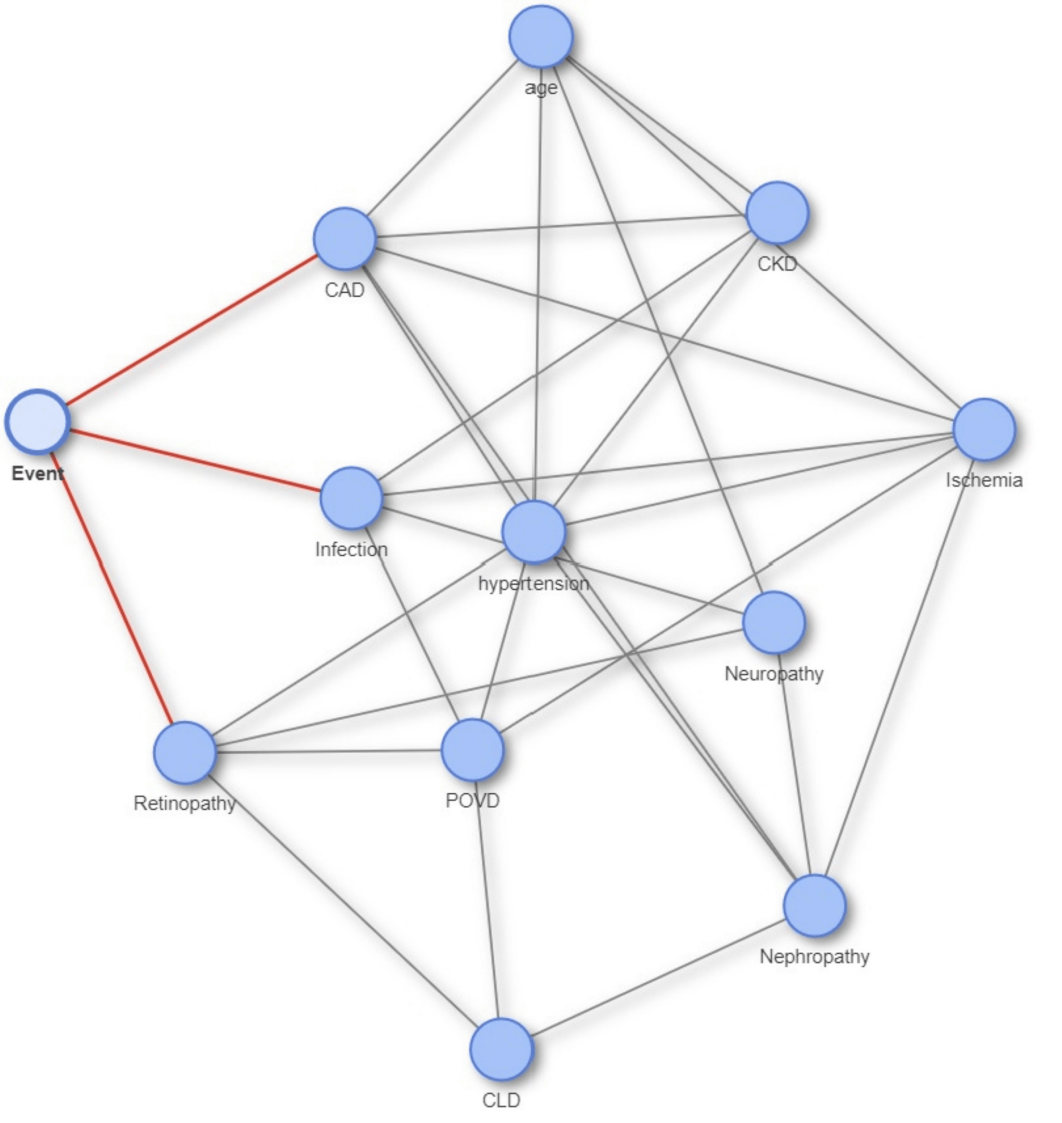
Bayesian network diagram to identify the potential confounders associated with the primary outcome CAD: coronary artery disease; CKD: chronic kidney disease; POVD: peripheral occlusive vascular disease; CLD: chronic liver disease Image credit: This is an original image created by the author Lloyson Benix

A Bayesian network diagram was created to identify the potential confounders associated with the outcome (Figure 2). It showed that infection, ischemia, CAD, CLD, neuropathy, and retinopathy were potential confounders affecting the time taken for wound healing.

To account for these differences, the remaining analysis would adjust for the potential confounders. Another logistic regression of the effect of HBOT on wound healing was performed while adjusting for the potential confounders identified from the Bayesian network. The results are presented in Table 3.

**Table 3:** Logistic regression of effect of HBOT on wound healing while adjusting for the potential confounders identified from the Bayesian network OR: odds ratio; CI: confidence interval; CAD: coronary artery disease; CLD: chronic liver disease; HBOT: hyperbaric oxygen therapy

Characteristic	OR	95% CI	p value
CAD	1.46	0.54, 4.43	0.5
CLD	1.55	0.20, 33.3	0.7
Retinopathy	0.24	0.05, 1.06	0.057
Neuropathy	0.97	0.25, 3.92	>0.9
Ischemia	0.80	0.28, 2.28	0.7
Infection	0.60	0.10, 2.81	0.5
University of Texas grade			
0	-	-	-
1	34.7	0.88, 1,570	0.044
2	3.15	0.11, 54.2	0.4
3	1.70	0.07, 22.4	0.7
Time	0.80	0.70, 0.91	0.001
Group			
Control	-	-	-
HBOT	2.32	0.57, 9.87	0.2

It included several patient characteristics as potential confounders. However, most of these characteristics, including CAD, CLD, neuropathy, ischemia, and infection, did not show a statistically significant association with wound healing (p > 0.5 for all). Retinopathy showed a marginally nonsignificant association with wound healing (p = 0.057). A wound severity of University of Texas grade 1 was found to be a significant predictor of wound healing (p = 0.044). Time also had a significant effect on wound healing (OR = 0.80, p = 0.001), indicating that the time to healing was longer in the HBOT group than in the control group because the HBOT group had a higher proportion of patients with severe wounds. While the OR for HBOT was 2.32, suggesting a positive effect, this did not reach statistical significance (p = 0.2). Even after adjusting for potential confounders, the effect of HBOT on wound healing showed a positive trend compared with standard-of-care treatment, but this effect was not statistically significant, likely owing to sample size limitations.

## Discussion

To date, various trials with adjunct HBOT treatment have not been able to demonstrate a consistent improvement in wound healing. Therefore, it has not been included as a standard-of-care treatment for chronic nonhealing diabetic ulcers. In our study, HBOT was administered as an adjuvant treatment only to patients with chronic nonhealing ischemic wounds, owing to which the experimental group patients had worse baseline ulcers than the control group patients.

Kranke et al. studied the impact of HBOT on chronic wounds, which included 10 trials in 531 participants with a diabetic foot ulcer. It showed an increase in the rate of ulcer healing with HBOT at six weeks, but this benefit was not evident at a longer term follow-up at one year [6]. Fedorko et al. conducted a randomized controlled trial (RCT) and concluded that HBOT did not provide an additional benefit over comprehensive wound care in reducing the indication for amputation or facilitating wound healing in patients with chronic diabetic foot ulcers [7].

A retrospective cohort study by Margolis et al. showed that HBOT did not significantly reduce amputation rates or improve the healing rates compared to standard wound care [8]. In a study performed by Salama et al., at the conclusion of an eight-week follow-up period, a considerably higher percentage of HBOT-treated wounds (33.3%) experienced full closure than wounds treated with conventional therapy (0%). However, the sample size was modest, with only 15 patients in each group [9].

According to a meta-analysis of 20 RCTs and 1,263 studies by Zhang et al., HBOT improved the healing rate of diabetic foot ulcers, shortened the healing period, and decreased the incidence of major amputations [10]. Sharma et al. did a meta-analysis of 14 studies, comprising 12 RCTs and two controlled clinical trials, which revealed that HBOT was ineffective for minor amputations and that the control group experienced fewer side effects. Additionally, there was no difference in the death rate or mean percentage of ulcer area reduction between the HBOT and control groups [11].

Stoekenbroek et al. concluded that larger, higher quality trials are required before the use of HBOT in general clinical practice for patients with diabetic foot ulcers can be justified [12]. Eleven RCTs comprising 668 patients were included in a study conducted by Moreira et al. After two weeks of treatment, patients receiving HBOT had a higher percentage of ulcer area reduction, lower risk of amputation, and higher likelihood of ulcer healing. There was no difference in the risk of minor amputations [13].

Oley et al. classified diabetic foot ulcers according to the Wagner grading system and demonstrated that supplementary HBOT delayed major and minor amputations in grade III and IV ulcers, prevented the need for debridement in grade II wounds, and enhanced wound healing for Wagner grades II, III, and IV [14]. According to Meethale Thiruvoth et al., HBOT reduced the percentage of minor and major amputations by 6.1% and 4.2%, respectively. They concluded that, in India, standard wound care, along with HBOT, had an incremental cost-effectiveness ratio of 193,939 Indian rupees per quality-adjusted life year gained, which was not economical [15]. In our study, each HBOT session cost 1,500 Indian rupees, with an average of 22,500 Indian rupees across the 15 sessions provided to the case group. This is not cost-friendly with regard to the socioeconomic status of our patients.

Considering the aforementioned studies on HBOT, there is no consistent evidence that HBOT is beneficial for healing diabetic foot ulcers, although it has been associated with reduced rates of minor or major amputations and shorter time to wound healing in some studies. The present study showed that HBOT improved the wound healing duration in ulcers of University of Texas grades 1, 2, and 3; however, it was found to be statistically significant only for grade 1 ulcers because of the small sample size in the HBOT group. However, the results are promising, and more trials are needed with a larger sample size and with fewer confounding factors to justify HBOT as a standard of care for treating chronic, nonhealing diabetic foot ulcers. The advantages of our study were that it included real-world data. Moreover, all patients were followed up for six months, which provided adequate time for complete wound healing. Furthermore, all patients in both the control and experimental groups received expert medical, surgical, and vascular management at a tertiary care setup; therefore, the observed impact of HBOT is reasonably reliable.

This study has certain limitations, as it is a nonrandomized retrospective study. Most severe ulcers (grade 3) were preferentially treated with HBOT, which introduced indication bias, which could limit comparability. Also, there is a possible confounding due to the nonuniformity in the management of ulcers in the case and control groups by the treating surgeon. However, because the entire study was conducted in the same department by the same treating team, the likelihood of differences in wound management is reduced, though it cannot be completely excluded. Moreover, the cost burden of HBOT and whether HBOT positively or negatively impacts the overall expenditure incurred until the ulcer is completely healed were not considered. Also, the significance of the number of HBOT sessions on wound healing was not examined. Future research addressing these limitations may justify including HBOT in the standard-of-care treatment for diabetic foot ulcers.

## Conclusions

This study showed a trend toward improved healing of diabetic foot ulcers with HBOT as an adjunct treatment. HBOT, when used as an adjunct to standard-of-care treatment, significantly shortened the healing time of University of Texas grade 1 ulcers. For more severe ulcers, HBOT demonstrated a positive trend toward wound healing, even though it was statistically insignificant. These findings suggest that HBOT can be used as an adjunct to standard-of-care dressings for healing diabetic foot ulcers without a substantial risk of adverse effects. Future RCTs and stratified studies in this regard with larger sample sizes and fewer confounding factors may enable us to justify including HBOT in the standard-of-care treatment for diabetic foot ulcers. Institutions can make policies to incorporate HBOT in diabetic foot ulcer management. Moreover, efforts should be made to make HBOT easily accessible for patients with diabetic foot ulcers. Widespread use of HBOT could improve diabetic foot ulcer healing and reduce the impending risk of amputation.
